# Retrieval of stuck burr by pullback orbital atherectomy with RESCUE technique: a case report

**DOI:** 10.1093/ehjcr/ytag259

**Published:** 2026-04-12

**Authors:** Sho Nakao, Takayuki Ishihara, Takuya Tsujimura, Wataru Ariyasu, Toshiaki Mano

**Affiliations:** Cardiovascular Center, Kansai Rosai Hospital, 3-1-69 Inabaso, Amagasaki, Hyogo 660-8511, Japan; Cardiovascular Center, Kansai Rosai Hospital, 3-1-69 Inabaso, Amagasaki, Hyogo 660-8511, Japan; Cardiovascular Center, Kansai Rosai Hospital, 3-1-69 Inabaso, Amagasaki, Hyogo 660-8511, Japan; Cardiovascular Center, Kansai Rosai Hospital, 3-1-69 Inabaso, Amagasaki, Hyogo 660-8511, Japan; Cardiovascular Center, Kansai Rosai Hospital, 3-1-69 Inabaso, Amagasaki, Hyogo 660-8511, Japan

**Keywords:** Rotational atherectomy, Burr stuck, Orbital atherectomy, Percutaneous coronary intervention, Case report

## Abstract

**Background:**

Rotational atherectomy (RA) burr entrapment is an uncommon but potentially life-threatening complication. Additional percutaneous bailout is necessary when conventional percutaneous bailout strategies are unsuccessful in patients with prohibitive surgical risk.

**Case Summary:**

A 91-year-old man with a history of coronary artery bypass graft surgery presented with unstable angina caused by severely calcified diffuse stenosis of the right coronary artery. During lesion preparation, an RA burr became entrapped after crossing a heavily calcified segment. Multiple conventional percutaneous bailout techniques, including guide-extension catheter support, intravascular lithotripsy for the stuck site, snare retrieval, and repeat RA, failed to retrieve the stuck burr. Surgical extraction was considered extremely high risk because of advanced age and prior cardiac surgery. Intravascular ultrasound revealed that the burr was embedded within a calcified nodule along the inner curvature. Orbital atherectomy (OAS) was subsequently advanced beyond the stuck site, and a low-speed pullback manoeuver successfully freed the burr, allowing safe retrieval. Final angiography and intravascular imaging confirmed adequate plaque modification and favourable luminal enlargement without major complications.

**Discussion:**

The pullback OAS with the REtrieval of StuCk burr by pUllback orbital athErectomy (RESCUE) technique can be a rational bailout option when the stuck RA burr is located along the inner-curvature calcification. Intravascular imaging assessment is essential for selecting this treatment strategy.

Learning points

- Intravascular imaging is essential for identifying the underlying mechanism of rotational atherectomy burr entrapment, and selecting an appropriate bailout strategy.
- Pullback orbital atherectomy may be a feasible percutaneous bailout technique in cases where a stuck burr is located along the inner curvature of heavily calcified lesions.

## Introduction

Rotational atherectomy (RA) burr entrapment is a characteristic complication occurring in approximately 0.5–1% of cases.^1–3^ Although infrequent, it can be fatal, making prompt and appropriate bailout essential. The Cardiovascular Intervention and Therapeutics (CVIT) consensus document proposes a percutaneous bailout algorithm for this complication;^3^ however, burr retrieval may still be challenging in certain situations, even with these techniques.

Herein, we present a case in which an additional atherectomy-based REtrieval of StuCk burr using the pullback orbital atherectomy (RESCUE) technique was successfully employed to retrieve a stuck RA burr.

## Summary figure

**Figure:**
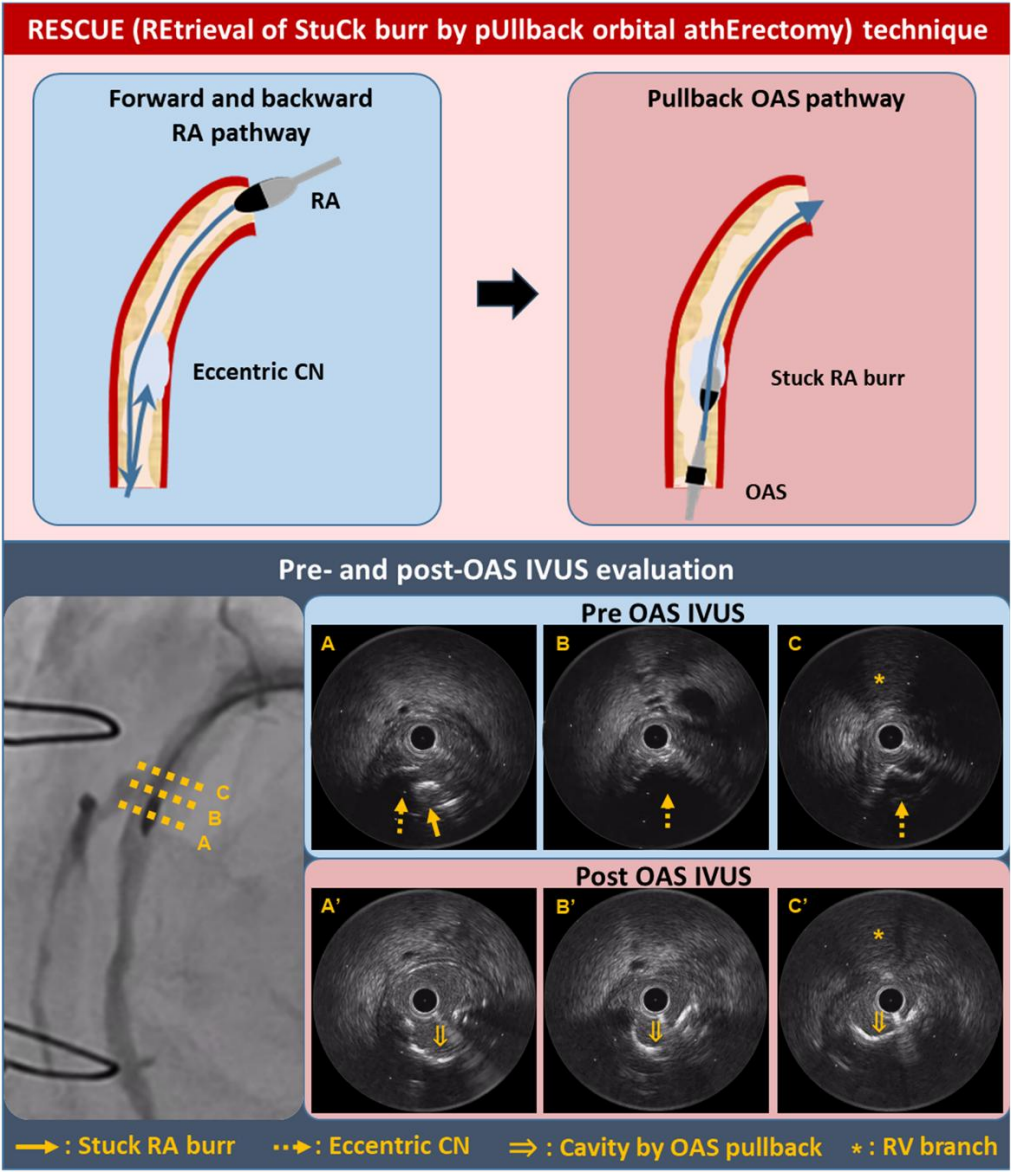


## Case presentation

A 91-year-old Asian man presented with dyspnoea on exertion and was diagnosed with congestive heart failure. Despite successful decongestion, the patient continued to experience chest discomfort, leading to coronary angiography for suspected unstable angina pectoris. Comorbidities included diabetes, hypertension, dyslipidemia, and chronic renal failure (estimated glomerular filtration rate: 35 mL/min/1.73 m^2^). The patient had undergone coronary artery bypass graft (CABG) surgery (left internal mammary artery to the left anterior descending artery and saphenous vein graft to the circumflex artery) more than 10 years prior. On physical examination, heart sounds were normal, and the patient was clinically euvolemic without peripheral oedema. His heart rate was 84 b.p.m., and systolic and diastolic blood pressures were 127 and 71 mmHg, respectively. Transthoracic echocardiography revealed a mildly reduced left ventricular ejection fraction (44%) primarily due to severe inferoposterior hypokinesis, along with moderate mitral regurgitation.

**Figure 1:**
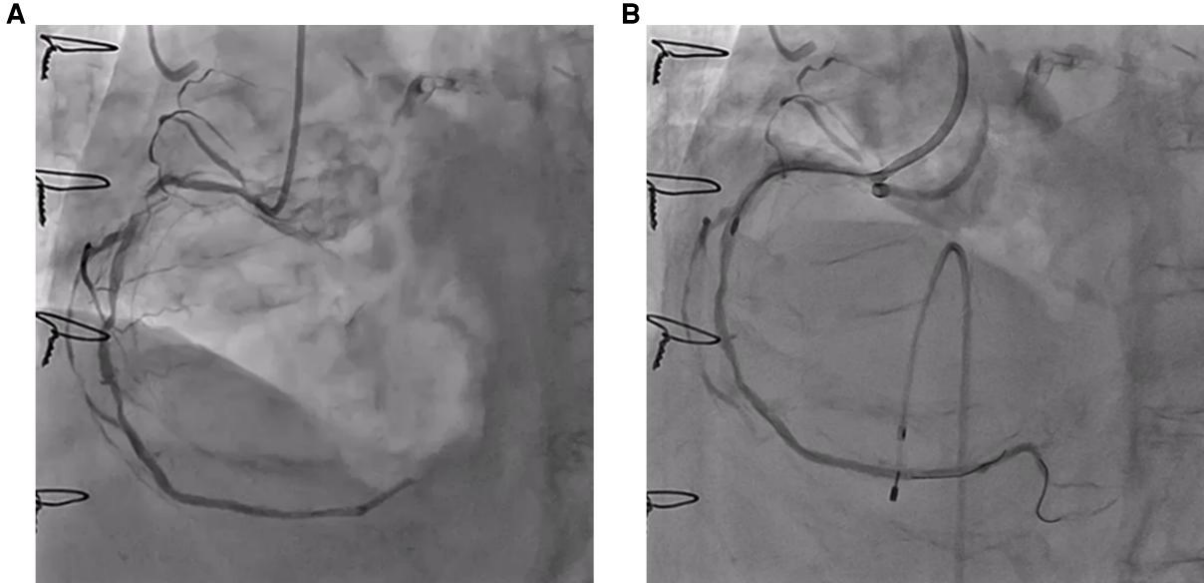
Initial and post-rotational atherectomy (RA) stuck burr angiography. (*A*) Initial coronary angiography reveals that the non-protected right coronary artery had severely calcified diffuse stenosis. (*B*) Although the RA burr suddenly jumped across the stenosis and became stuck, the coronary flow is preserved at the time of burr entrapment.

Coronary angiography showed patent bypass grafts and severely calcified, diffuse stenosis of the right coronary artery (RCA) (*Figure 1*, Supplementary material online, *Video S1*), which was selected for percutaneous coronary intervention. A 7Fr short Amplatz Left 1 guide catheter was introduced through the right radial artery. After crossing the lesion with a SION Blue wire (Asahi Intecc, Irvine, CA, USA), intravascular ultrasound (IVUS) was attempted but could not be advanced because of severe proximal calcification. Therefore, we proceeded with RA. After exchanging for a Rota floppy wire (Boston Scientifics, Marlborough, MA, USA), RA was initiated with a 1.5-mm burr at 180 000 rpm. However, the burr could not cross the ostial calcified lesion. We then stepped down to a 1.25-mm burr (180 000 rpm) to modify the proximal calcified lesion, followed by debulking of the most stenotic lesions. On the eighth attempt, the burr suddenly jumped across the stenosis, immediately stalled, and became entrapped (see Supplementary material online, *Video S2*). Fortunately, coronary flow was preserved (Thrombolysis in Myocardial Infarction [TIMI] grade 3) (*Figure 1*).

Gentle manual traction on the RA wire and burr was attempted but unsuccessful. The burr shaft was then cut, the driveshaft sheath removed, and multiple guide-extension catheters (GECs), 7Fr GuideZilla (Boston Scientifics), 6Fr, and 5Fr Guideplus (Nipro, Osaka, Japan) were attempted to strengthen localized withdrawal force. However, none could be advanced because of severe proximal calcification, even after dilation using a 2.5-mm non-compliant balloon. Subsequently, to facilitate further modification of the proximal calcification with intravascular lithotripsy (IVL), an 8Fr short Amplatz Left 1 guide catheter was inserted via the right common femoral artery. After 80 pulses using a 2.5-mm Shockwave C2^+^ catheter (Shockwave Medical, Santa Clara, CA, USA) from the site of the burr stuck to the RCA ostium, a 6Fr GEC could finally be delivered just proximal to the stuck burr. However, the burr remained completely immobile. We then attempted snare-assisted traction using a 4.0-mm Amplatz GooseNeck snare (Medtronic, Minneapolis, MN, USA). This approach also failed, suggesting that the burr was firmly embedded within a focal calcified structure. Although surgical removal was considered, the patient’s advanced age and previous cardiac surgery prompted us to pursue alternative percutaneous strategies.

**Figure 2:**
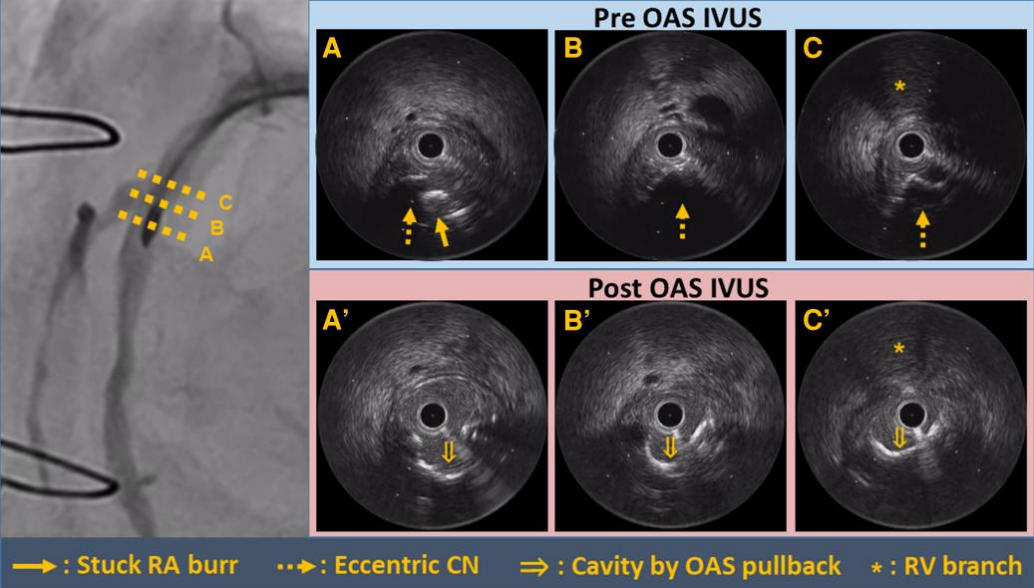
Pre- and post-orbital atherectomy system (OAS) intravascular ultrasound (IVUS) evaluation. (*A*)–(*C*) Pre-OAS IVUS demonstrates that the rotational atherectomy (RA) burr is lodged within an eccentric calcified nodule (CN) on the inner curvature of the vessel (the opposite side of the right ventricular [RV] branch). The yellow solid arrow indicates the RA burr. Yellow dashed arrows indicate the CN. Yellow asterisks indicate the RV branch. (*A*′)–(*C*′) After OAS, IVUS confirmed adequate modification of the CN, with cavity formation and significant lumen enlargement. Yellow double arrows indicate the enlarged lumen cavity.

In the next step, we performed IVUS to clarify the mechanism of burr entrapment, which revealed diffuse, circumferential calcification, and the burr lodged within an eccentric calcified nodule (CN) on the vessel’s inner curvature (*Figure 2*, Supplementary material online, *Video S3*). Based on these imaging findings, further traction-based strategies were considered unlikely to succeed, and an atherectomy-based approach to modify the inner-curvature calcification was selected. First, another RA with a 1.5-mm burr was carefully attempted using a support wire. Although the burr passed beyond the stuck site, it remained immobile.

Thereafter, orbital atherectomy (OAS) was performed using the Diamondback 360 Coronary Orbital Atherectomy System (CSI, St. Paul, MN, USA). We successfully delivered the OAS beyond the stuck site using the GlideAssist mode. During the first low-speed pullback run (80 000 rpm, with a duration of <10 s), the stuck burr moved slightly proximally, enabling successful retrieval (see Supplementary material online, *Video S4*). High-speed mode was not used because bailout was achieved during the initial low-speed pullback run. Subsequent angiography showed transient TIMI grade 2 flow, which improved to TIMI grade 3 after intracoronary nitroglycerin administration. IVUS confirmed adequate modification of the CN with cavity formation and significant lumen enlargement without visible calcium fracture. The minimal lumen area on IVUS after RA burr stuck was 2.58 mm^2^, which increased to 3.62 mm^2^ following OAS (*Figure 2*, Supplementary material online, *Video S5*). The procedure was completed using two overlapping drug-coated balloon inflations (Agent 2.5 × 30 mm; Boston Scientifics), resulting in an angiographically optimal outcome with TIMI grade 3 flow (see Supplementary material online, *Video S6*). The patient’s subsequent course was uneventful, with no significant elevation in creatine kinase levels, and he was discharged promptly. Following the procedure, the patient experienced improvement in angina symptoms and was relieved by the favourable clinical outcome.

## Discussion

The CVIT consensus document recommends a stepwise algorithm for managing RA burr entrapment.^3^ The first step involves balloon dilatation at the site of burr entrapment, often combined with adjunctive manoeuvres such as cutting the driveshaft, removing the sheath, or introducing a second guide catheter. The second step involves deep intubation of the guide catheter, a second smaller guiding catheter, or GEC, followed by simultaneous traction on the RA system and counter-traction on the guide catheter. Additional techniques, such as snare use or balloon trapping within the guide catheter, may increase localized withdrawal force. If these percutaneous strategies fail, surgical burr removal is recommended.

Despite these strategies, additional percutaneous bailout may be required in selected cases, particularly when surgical risk is prohibitive due to advanced age, prior thoracotomy, immunocompromise, or multiple comorbidities. In the present case, almost all conventional bailout techniques were unsuccessful, and surgical extraction was deemed extremely high risk owing to advanced age and prior CABG history.

Several alternative bailout techniques for rotational atherectomy burr entrapment have been reported. Maznyczka *et al*. reported the ‘LAST (Limited Antegrade Subintimal Tracking) technique,’ which involves subintimal entry proximal to the burr to externally crush plaque to disengage the burr.^4^ However, this approach requires intentional subintimal entry and re-entry into the true lumen using a high-gram-tip wire, which can be technically demanding and may increase the risk of vessel perforation, particularly in elderly patients with diffusely calcified as in the present case. IVL has also been an adjunctive method to facilitate burr retrieval by modifying calcified plaque.^5^ In this case, IVL was useful for enabling GEC delivery; however, it was insufficient to release the burr, likely because the burr was firmly embedded within an eccentric calcified nodule rather than constrained by concentric sheet calcification alone. Stenting over the burr *in situ* (crush technique) has been described as an ultimate bailout option when the burr or the Rotawire becomes detached and retrieval is impossible.^6^ Nevertheless, this approach generally requires acceptance of permanent stent implantation over an incompletely prepared, heavily calcified lesion and may result in suboptimal stent expansion; therefore, it was considered a last-resort option in our case.

From a mechanistic standpoint, RA burr entrapment in this case can be explained by the interaction between vessel curvature, eccentric calcification, and the intrinsic tracking behaviour of the RA system. The structural characteristics of the oval-shaped RA burr—featured by a diamond-encrusted distal portion and a smooth proximal portion without sputtering—allows plaque modification during forward advancement but not during pullback.

Additionally, the burr tends to align along the outer curvature during advancement and shifts toward the inner curvature during withdrawal, predisposing it to entrapment within eccentric calcification along the inner curve. In the present case, incomplete modification of the proximal calcified segment allowed the burr to abruptly jump distally and, on withdrawal, become embedded within a calcified nodule located along the inner curvature, as clearly demonstrated by IVUS. This configuration created a mechanical lock that rendered conventional traction-based bailout techniques ineffective.

We utilized the ‘RESCUE technique’ to modify the severely calcified plaque using OAS. The OAS features a 1.25-mm crown that rotates in orbital motion, debulking plaque during both forward and backward movements. This enables effective modification of calcification along the inner curvature on withdrawal, precisely where the RA burr tends to become stuck. Indeed, IVUS after OAS demonstrated erosion of the CN along its inner side facilitating burr release.

In the present case, IVUS was pivotal for defining the underlying mechanistic perspective of RA burr stuck and selecting the bailout strategy. IVUS identified the burr embedded within a protruding eccentric calcified nodule along the inner curvature, suggesting that successful release required targeted modification of inner-curvature calcium. Therefore, pullback OAS provided a mechanistically rational and effective bailout strategy, although its risks must be carefully considered.

The potential complications include vessel injury, RA wire fracture, and burr detachment. Advancing an OAS crown beyond a trapped RA burr may increase the risk of vessel perforation, particularly in severely calcified and tortuous segments, and mechanical interaction between the rotating OAS crown and the stationary RA burr may theoretically increase the risk of device damage or wire fracture; therefore, this bailout should be attempted only by highly experienced operators, ideally after confirming haemodynamic stability (with circulatory support considered when appropriate). During the OAS pullback, retracting the RA wire to the spring tip, where the wire diameter increases, reduces the risk of wire fracture. Even if fracture occurs, stent implantation over the disrupted wire remains feasible after burr retrieval. Burr detachment is another concern; in such cases, crush technique represents the ultimate bailout strategy.^5^

## Conclusion

Our case demonstrates that pullback OAS can serve as a valuable percutaneous bailout strategy alongside conventional techniques. Intravascular imaging assessment is essential for identifying the underlying mechanism of RA burr stuck. The RESCUE technique is particularly effective when a stuck burr is located along the inner curvature of the calcification.

## Lead author biography


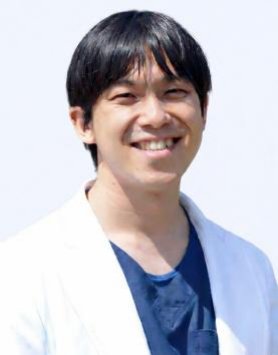


Sho Nakao, MD, is an interventional cardiologist at Kansai Rosai Hospital, Japan, and is scheduled to undertake a postdoctoral research fellowship at Mount Sinai Hospital, New York, USA. His clinical and research interests include complex coronary intervention, plaque modification strategies such as atherectomy and intravascular imaging. He has published several articles in peer-reviewed journals and is committed to advancing minimally invasive strategies for high-risk coronary artery disease.

## Supplementary Material

ytag259_Supplementary_Data

## Data Availability

The data that support the findings of this study are available from the corresponding author upon reasonable request.

## References

[1] Morita Y, Kashima Y, Yasuda Y, Kanno D, Hachinohe D, Sugie T, et al Burr entrapment in a percutaneous coronary intervention during rotational atherectomy: an experience with 3195 cases. J Invasive Cardiol 2023;35. 10.25270/jic/23.00174 37984323

[2] Sulimov DS, Abdel-Wahab M, Toelg R, Kassner G, Geist V, Richardt G. Stuck rotablator: the nightmare of rotational atherectomy. EuroIntervention 2013;9:251–258. 23793010 10.4244/EIJV9I2A41

[3] Ogawa T, Sakakura K, Sumitsuji S, Hyodo M, Yamaguchi J, Hirase H, et al Clinical expert consensus document on bailout algorithms for complications in percutaneous coronary intervention from the Japanese association of cardiovascular intervention and therapeutics. Cardiovasc Interv Ther 2025;40:1–32. 39627466 10.1007/s12928-024-01044-y PMC11723903

[4] Maznyczka A, Mozid A. The limited antegrade subintimal tracking technique to retrieve a trapped rotablator burr: a case report. Eur Heart J Case Rep 2024;8:ytae044. 38328602 10.1093/ehjcr/ytae044 PMC10849080

[5] Ban S, Yamamoto K, Mase T, Arao K. A novel bailout method for rotational atherectomy burr entrapment using intravascular lithotripsy. Cardiovasc Interv Ther 2026 Jan 20. doi: 10.1007/s12928-026-01241-x. Epub ahead of print. 41557083

[6] Boueri Z, Mazloum R, Riccini P, Souteyrand G, Amabile N. Crush technique as ultimate bailout treatment for rotablator burr entrapment. JACC Case Rep 2025;30:103129. 39963206 10.1016/j.jaccas.2024.103129 PMC11830266

